# InSiDDe: A Server for Designing Artificial Disordered Proteins

**DOI:** 10.3390/ijms19010091

**Published:** 2017-12-29

**Authors:** Antoine Schramm, Philippe Lieutaud, Stefano Gianni, Sonia Longhi, Christophe Bignon

**Affiliations:** 1 Aix-Marseille University, CNRS, Architecture et Fonction des Macromolécules Biologiques (AFMB), UMR 7257 Marseille, France; Antoine.Schramm@afmb.univ-mrs.fr (A.S.); Philippe.lieutaud@afmb.univ-mrs.fr (P.L.); 2 Istituto Pasteur Italia-Fondazione Cenci Bolognetti, Istituto di Biologia e Patologia Molecolari del CNR, Dipartimento di Scienze Biochimiche “A. Rossi Fanelli”, Sapienza Università di Roma, 00185 Rome, Italy; stefano.gianni@uniroma1.it

**Keywords:** intrinsically disordered proteins, artificial protein, IUPred, recombinant protein, Python3, paramyxovirus, N_TAIL_, XD, *E. coli*

## Abstract

InSiDDe (In Silico Disorder Design) is a program for the in silico design of intrinsically disordered proteins of desired length and disorder probability. The latter is assessed using IUPred and spans values ranging from 0.55 to 0.95 with 0.05 increments. One to ten artificial sequences per query, each made of 50 to 200 residues, can be generated by InSiDDe. We describe the rationale used to set up InSiDDe and show that an artificial sequence of 100 residues with an IUPred score of 0.6 designed by InSiDDe could be recombinantly expressed in *E. coli* at high levels without degradation when fused to a natural molecular recognition element (MoRE). In addition, the artificial fusion protein exhibited the expected behavior in terms of binding modulation of the specific partner recognized by the MoRE. To the best of our knowledge, InSiDDe is the first publicly available software for the design of intrinsically disordered protein (IDP) sequences. InSiDDE is publicly available online.

## 1. Introduction

Intrinsically disordered proteins (IDPs) or regions (IDRs) lack a defined secondary and tertiary structure under physiological conditions of pH and salinity in the absence of a binding partner or ligand [1,2,3,4,5,6,7]. IDPs span a large structural diversity due to various levels of compaction and of transiently populated secondary structures [1,2,3,4,5,6,7,8].

Many IDPs undergo a disorder-to-order transition upon binding to their partner(s) [6,7]. This structural transition is often limited to a short region, referred to as molecular recognition element (MoRE) [9], whilst the rest of the protein remains “fuzzy” [10], retaining a large conformational entropy. In an effort aimed at elucidating the impact of fuzzy appendages on partner recognition, we have previously investigated the impact of the fuzzy appendage of the intrinsically disordered C-terminal domain of the nucleoprotein (N_TAIL_) of three paramyxoviruses on binding to the structured X domain (XD) of the homologous phosphoprotein. Among these IDRs, the measles virus (MeV) N_TAIL_ is made of an N-terminal fuzzy region (amino acids 401 to 485) followed by a MoRE (amino acids 486 to 502). We showed that the fuzzy appendage acts as a natural dampener of the N_TAIL_/XD interaction, with gradual shortening of the fuzzy appendage leading to increased interaction strength [11]. To determine if the dampening effect was sequence-dependent or not, we designed an artificial fuzzy appendage (amino acids 401 to 481) with a low sequence identity with the wild-type (wt) sequence and a higher disorder score as assessed using IUPred [12]. When the wt and the artificial fuzzy appendages were swapped within full length N_TAIL_, the artificial N_TAIL_ protein was successfully expressed in *E. coli* and its fuzzy region was found to inhibit the interaction with XD in a way very similar to that of wtN_TAIL_ [11]. In addition, the gradual N-terminal shortening of the artificial region led to the same pattern as observed with wtN_TAIL_, that is, the interaction with XD increased with the shortening. These results allowed us to conclude that the effect of N_TAIL_ fuzzy appendage on XD binding mainly depended on the length of the fuzzy appendage and not on a specific sequence [11].

Results reported in that study definitely indicated that it was possible to devise an artificial IDP that could be expressed in *E. coli* as a recombinant protein. However, the design of an artificial protein in the absence of a dedicated tool proved to be time and labor consuming. In spite of the interest in IDPs in the last decade, our search for published methods or online servers intended for ex nihilo generating artificial IDP proved unsuccessful. Previous studies made use of multiple glycine substitutions to convert an α-helical region into a disordered region (for examples see [13,14]). A recent study reported the in silico generation of artificial IDPs [15] but no details were provided on how the sequences were conceived and no mention was made of a possible fully automated, publicly available server specifically for this. Previous elegant studies by the group of Rohit Pappu reported the development of GADIS, a method for designing IDP sequences of specified intrinsic helicities while maintaining the native amino acid composition and specific residues at the binding interface [16]. However, all these studies make use of a pre-existing protein sequence. This prompted us to devise our own method for in silico generation of artificial IDPs, independently of any pre-existing protein sequence. To that end, we setup InSiDDe (In Silico Disorder Design) a server for designing amino acid sequences of a desired length and predicted disorder score. Here, we describe the rationale we used and present results showing that a sequence designed using InSiDDe and fused to the MoRE of MeV N_TAIL_ could be recombinantly expressed in *E. coli* at high levels without degradation. Sequences had features typical of an IDP and exhibited the expected behavior in terms of modulation of binding strength towards MeV XD, the natural partner recognized by the MoRE.

## 2. Results and Discussion

### 2.1. Rationale and Implementation of In Silico Disorder Design (InSiDDe)

InSiDDe relies on the generation of a library of sequences of 10 residues and then on the subsequent use of this library to generate artificial sequences.

#### 2.1.1. Creation of the Sequence Library

**Figure 1:**
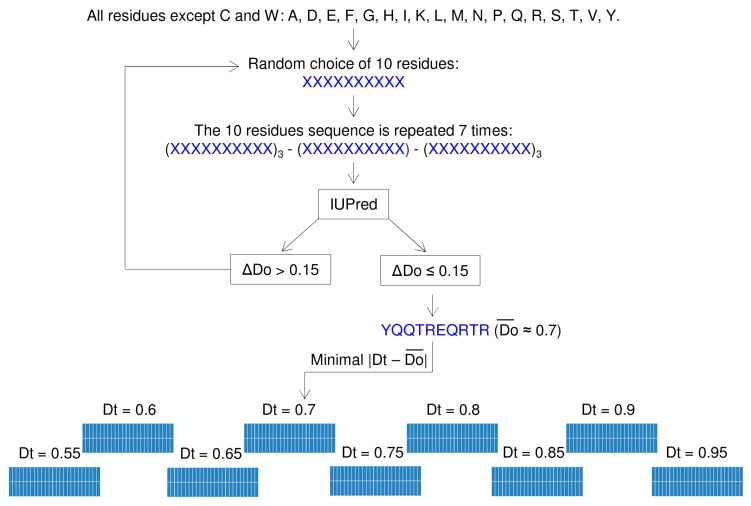
Creation of the bb library. An example with a target disorder probability (*D*t) = 0.7 is displayed. The nine families, each containing 50 building blocks (bbs), are represented by gridded blue rectangles with *D*t values indicated above.

**Figure 2:**
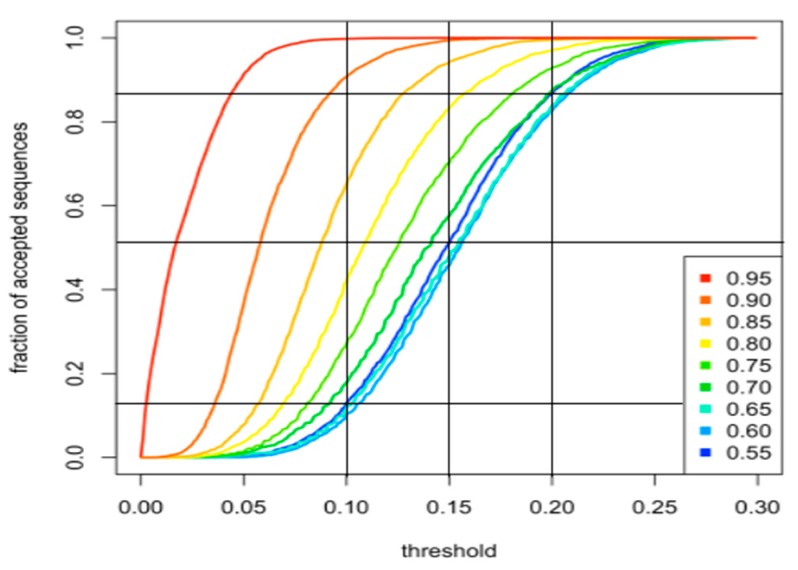
Choice of bb ∆*D*o value for each rejection threshold. The fraction of accepted bb sequences (y-axis) is reported as a function of the threshold value for each *D*t (x-axis). Vertical lines locate 0.1, 0.15 and 0.2 thresholds on the curves. The three horizontal lines cross each vertical line on one of the three *D*t curves with the lowest *D*t values (i.e., 0.55, 0.60, 0.65). Bbs were generated as described in Figure 1. Inset, color code of *D*t values.

Initially, a library of “building blocks” (bbs), each made of 10 residues, was generated (Figure 1). Building blocks were clustered into nine families, each containing 50 bbs. The nine families had a target disorder probability (*D*t) ranging from 0.55 to 0.95 with 0.05 increments. The average observed disorder probability (*D*o) of each bb was assessed using IUPred. The rationale for setting these Dt boundaries was that 0.5 is the IUPred threshold above which a sequence is considered as disordered and 1.0 is the highest IUPred disorder score [12]. IUPred was chosen because we had already successfully used it to analyze the artificial N_TAIL_ sequence designed and experimentally characterized in our previous study [11] and because it could be run in local mode on our server. However, this choice does not exclude that other programs or meta-programs could have been used. bbs were generated by randomly choosing 10 amino acids in a pool from which W and C had been eliminated. Indeed, including these two residues systematically imparted an average IUPred disorder score below 0.55 because W is the most order-promoting residue [17] and C has the potential to stabilize tertiary structure through the formation of disulphide bonds. Each of the remaining 18 amino acids had an equal probability of being chosen and amino acids were sampled with replacement. The sequence of the first bb was repeated seven times and the disorder propensity of the central bb analyzed using IUPred. There were several reasons why this number of repeats was chosen. To estimate the position-specific energies of the sequence under study (in our case, a 10-residues bb), IUPred uses a sliding window of 21 residues. To avoid any risk of fringe effects, we chose to use a large excess (i.e., 3 bbs) of N- and C-terminal residues, hence the seven times repetition. Then, the absolute value of the difference between each of the nine target disorder probability values and the mean observed disorder score (|*D*t − $\bar{Do}$|) was calculated, as was the absolute value of the difference between the maximum and the minimum observed disorder score (Δ*D*o = |*D*o_max_ − *D*o_min_|) of the central 10 residues. |*D*t − $\bar{Do}$| measures the agreement of the IUPred result with one of the nine target disorder probability values and allows InSiDDe to assign the bb to the proper familly. Δ*D*o measures the smoothness of the curve (the lower the Δ*D*o, the smoother the disorder probability profile). If Δ*D*o > 0.15, the block is discarded and another bb is randomly assembled. If Δ*D*o ≤ 0.15, the block is accepted and stored in the family of bbs with the closest *D*t. The value of 0.15 was chosen because it allowed the highest percentage of generated bbs to be accepted whatever the bb *D*t value while maintaining an acceptable smoothness. As can be seen in Figure 2, the bbs with the three lowest *D*t values (0.55, 0.60, 0.65) were the most prone to rejection as a function of the Δ*D*o threshold value. With Δ*D*o > 0.2, almost 90% of sequences with these *D*t values were accepted but at the price of bad smoothness. Conversely, with Δ*D*o threshold of 0.10, the smoothness was improved but only about 15% of low *D*t value bbs were accepted. Incidentally, we have no explanation for the observed high rejection rates of low *D*t bbs compared to high *D*t bbs. A threshold value of 0.15 for Δ*D*o was therefore chosen as an acceptable tradeoff. The entire procedure was repeated until each of the nine families contained 50 bbs.

**Figure 3:**
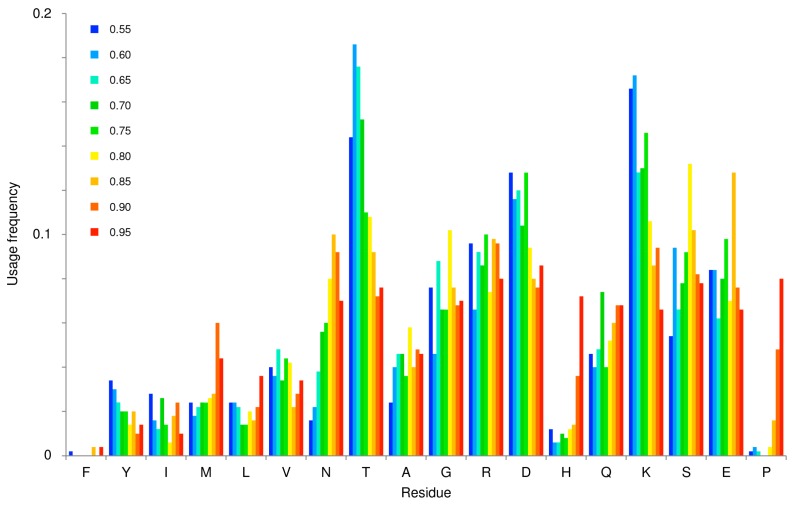
Amino acids usage frequency in the bb library. Shown is the usage frequency (y-axis) of each of the 18 amino-acids (i.e., excluding W and C) used by each bb family (the color code of which is indicated on the left of the graph) in the library. Residues are sorted from left to right on the x-axis by increasing disorder propensity according to the TOP-IDP scale [17].

In Figure 3, we analyzed the usage frequency by InSiDDe of the 18 residues in the bb library. No simple rule can be inferred from this graph but basically it can be argued that, except for N and T, the nine most order-promoting residues (from F to A) tend to be less used than the nine most disorder-promoting residues (except for H and P). Interestingly, the two extremes of the x-axis (F and P) are almost never used. In other words, InSiDDe seems to avoid using the least and the most disorder-promoting residues while favoring disorder promoting residues. The overall trend probably reflects the *D*t space in which InSiDDe is allowed to work (from 0.55 to 0.95) and the specific purpose for which it was built, i.e., generating disordered sequences. It is likely that including folded proteins (i.e., a 0 to 0.95 working range instead of 0.55 to 0.95) would have provided a more balanced profile with a higher usage frequency of more order promoting residues.

#### 2.1.2. Generation of Artificial Sequences

**Figure 4:**
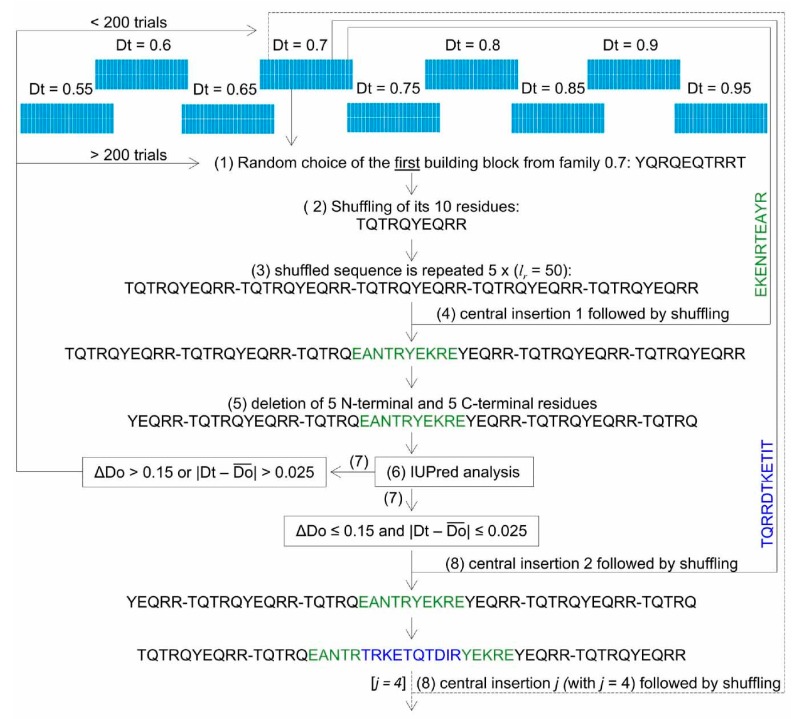
Generation of artificial intrinsically disordered proteins (IDP) by In Silico Disorder Design (InSiDDe). In this example, an IDP with |$\bar{Do}$| = 0.7 and *l_r_* = 50 is displayed. The nine families of building blocks are represented as in Figure 1. Numbers in parentheses are the same as those used in the corresponding part of the Results and Discussion section.

Using the above described library, InSiDDe generates artificial amino acid sequences with specified lengths (*l*) and *D*t through the following steps (Figure 4): (1) random choice of one out of the 50 bbs of the specified *D*t family; (2) shuffling of its 10 residues; (3) generation of a sequence of length *l_r_* (with *l_r_* corresponding to the desired final length *l* rounded to the next upper multiple of 10) by repetition of this block *i* times (with *i* = *l_r_*/10); (4) insertion in the middle of the sequence of another randomly chosen bb from the same Dt family followed by shuffling of the bb sequence; (5) deletion of the five N-terminal and five C-terminal residues to keep *l_r_* constant; (6) analysis by IUPred of the new sequence; (7) conservation or rejection of the new sequence (see below); (8) repetition of steps 4 to 7 *j* times (with *j* = (*l_r_*/10) − 1); and (9) deletion of the extra residues in order to obtain the desired sequence length if *l* is not a multiple of 10. In the latter case, the final sequence of length *l_r_* provided by the program is shortened to match the desired length *l* by removing the appropriate number of residues from the extremity displaying the most dissimilar disorder score with respect to the chosen *D*t.

**Figure 5:**
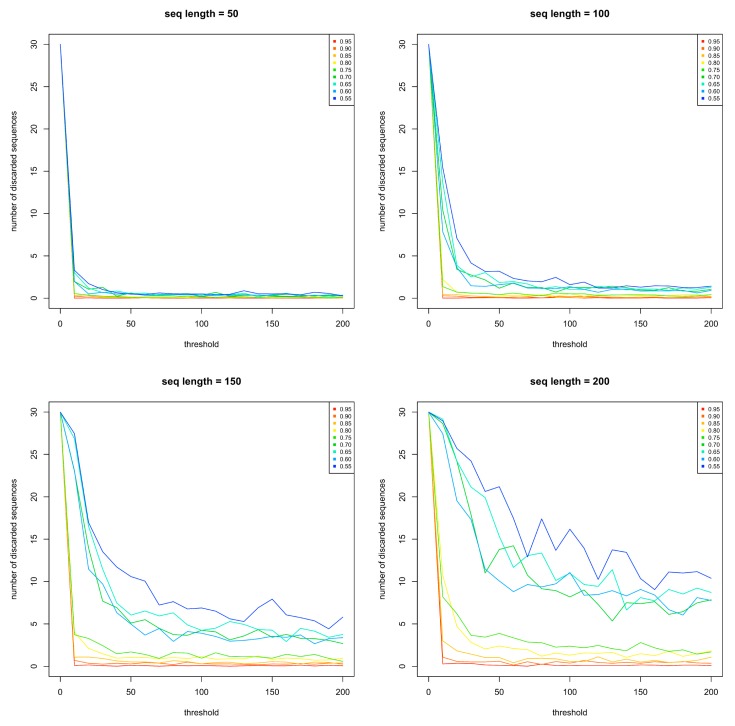
Choice of central bb insertion iteration value for each rejection threshold. The number of rejected sequences (y-axis) was calculated for different sequence lengths (50 residues (top left subfigure), 100 residues (top right subfigure), 150 residues (bottom left subfigure), 200 residues (bottom right subfigure)), different rejection thresholds (x-axis), and different *D*t (the color code of which is indicated in an inset in each subfigure).

After each central insertion of a bb, the disorder propensity of the entire new sequence is computed using IUPred, as described above in steps 4 to 6. If Δ*D*o ≤ 0.15 and |*D*t − $\bar{Do}$| ≤ 0.025, the sequence is accepted for the next step. If Δ*D*o > 0.15 or |*D*t − $\bar{Do}$| > 0.025, the central insertion of 10 residues is discarded and a new sequence is generated by inserting in the middle of the sequence another bb randomly chosen in the same family. At each central insertion, the maximum number of rejections was set to 200. This threshold was chosen because it confidently provided the minimal number of discarded sequences, as can be seen in Figure 5. Under the most computationally demanding conditions (sequence length = 200 residues, *D*t = 0.55), no further failure rate decrease was discernable between thresholds 150 and 200. As expected, Figure 5 shows an increased failure rate with increasing sequence length and decreasing threshold values. However, we cannot explain the failure rate increase with decreasing *D*t already observed during the generation of bbs and shown in Figure 2. Above 200 failed insertion trials, the whole sequence is discarded and a new initial bb is randomly chosen (i.e., the generation of the artificial sequence is aborted).

**Figure 6:**
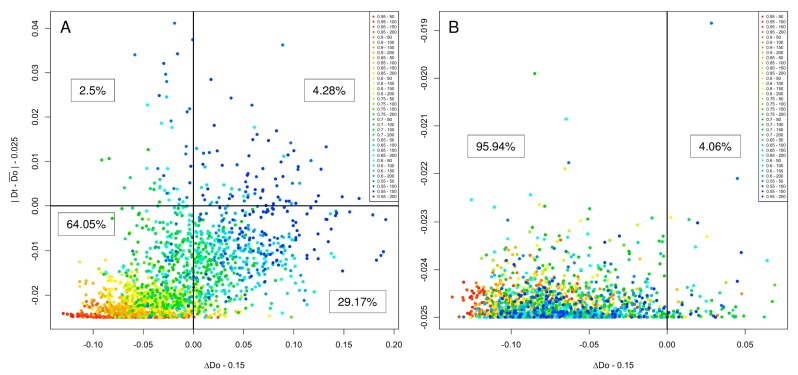
Comparison between two alternative methods for generating the starting IDP sequence. For each generated sequence, ∆*D*o and |*D*t − $\bar{Do}$| were computed and subtracted from their corresponding threshold (0.15 and 0.025, respectively, as indicated on the x- and y-axes). Sequences are accepted by InSiDDe only if (∆*D*o − 0.15) and (|*D*t − $\bar{Do}$ | − 0.025) are both negative. (**A**) The first sequence (step (1) in Figure 4) was generated by appending randomly chosen bbs from one family of the bb library; (**B**) The first sequence (step (1) in Figure 4) was generated by repeating a randomly chosen bb from one family of the bb library. The percentage of sequences is indicated for each combination of positive or negative (∆*D*o − 0.15) and (|*D*t − $\bar{Do}$ | − 0.025) values. The color code of each combination of *D*t values (ranging 0.55 to 0.95) and sequence lengths (50, 100, 150 and 200 residues) is indicated in the box on the right of each graph.

The rationale for generating an initial sequence made of a repetition of the same bb, rather than of different bbs from the same *D*t family, lies in the requirement to start with a sequence with the smallest |*D*t − $\bar{Do}$| and Δ*D*o values. In a repeated sequence, the interaction between a given amino-acid and the others is constant through the entire length. As IUPred score is based on the interactions between a given residue and the adjacent ones in a defined window, a sequence based on the repetition of the same bb is the easiest way to produce an initial sequence satisfying these criteria. This expectation was confirmed by performing the experiments reported in Figure 6. In Figure 6A, the initial sequence was generated by associating several bbs chosen at random in the same *D*t family. In Figure 6B, the initial sequence was generated by repeating the same bb chosen at random in a given *D*t family. Comparison of Figure 6A,B definitely indicates that the second strategy generated much more acceptable sequences (≈96%) than the first (≈64%). One might question why further development of the program is necessary if the mere repetition of a bb is enough to create an artificial protein with the required |*D*t − $\bar{Do}$| and Δ*D*o. A repetitive protein sequence would also be repetitive at the DNA level, a feature that should be avoided because repetitive DNA sequences tend to recombine in vivo, which eventually produces deleted sequences [18]. In addition, we wanted to generate sequences with as much diversity as possible. For these reasons, InSiDDe was conceived to avoid long repetitive sequences. Note that the shuffling step (Figure 1 and Figure 4) follows the same rationale: it increases diversity by reducing repetition among the same generated sequence.

**Figure 7:**
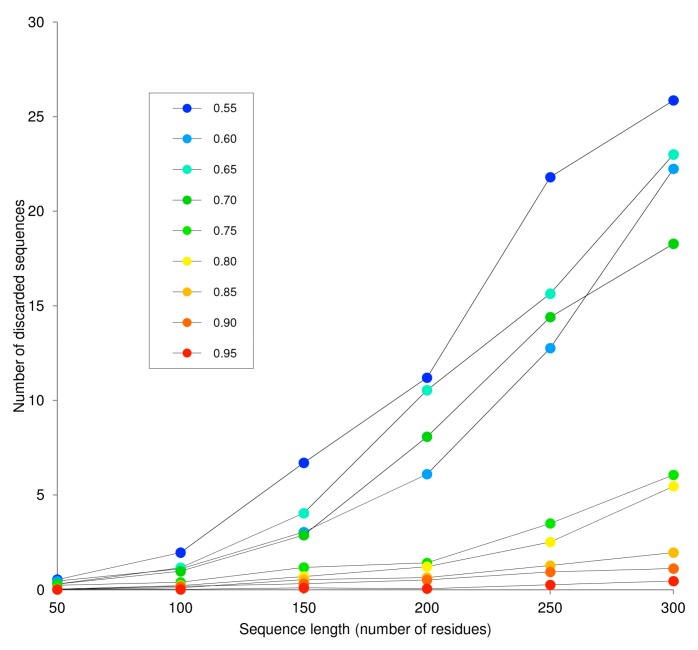
Number of discarded sequences as a function of *l_r_* and *D*t. Each experimental point is the mean value obtained from 50 independent InSiDDe requests. See the Results and Discussion section and Figure 4 for details.

The length of artificial sequences generated by InSiDDe ranges from 50 to 200 residues. The lower limit is dictated by IUPred specificities and the upper limit by the connection time out per sequence that has been set to 5 min. To assess the largest sequence length that can be generated before this time threshold is reached, we calculated the number of discarded sequences, as a function of sequence length, for different *D*t values. As expected, Figure 7 clearly shows an increase in the number of discarded sequences with increasing *l_r_* and, hence, in the time required to generate a sequence. However, Figure 7 also shows an unexpected increase in the number of discarded sequences when *D*t decreases, as already seen in the experiments reported in Figure 2 and Figure 5. We have no explanation for this observation since the sequences are generated starting from the same number of bbs (i.e., 50) whatever their *D*t. According to Figure 7, if sequences exceeding 200 amino acids can be quickly obtained by InSiDDe provided that they have a high IUPred disorder score (i.e., *D*t > 0.75), generating lengthy sequences with low *D*t values is more prone to failure. For that reason, the longest sequence computed by InSiDDe has been set to 200 residues.

**Figure 8:**
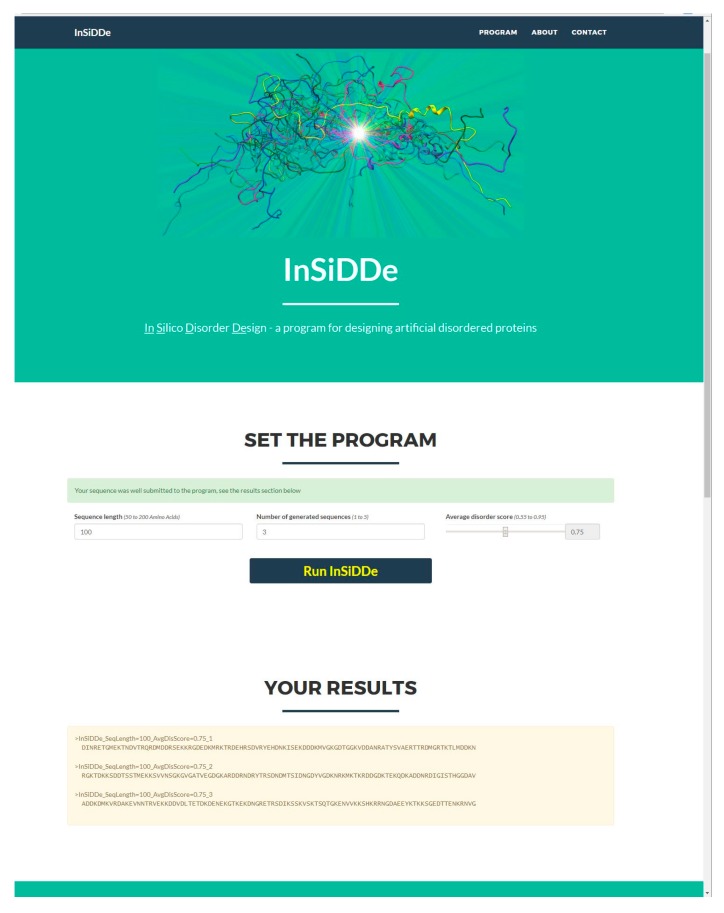
Snapshot of the web interface of InSiDDe.

InSiDDe is publicly available at http://insidde.afmb.univ-mrs.fr/. Figure 8 shows a screen-capture of the web interface. Under the display banner “SET THE PROGRAM”, the user is prompted to (i) enter the length of the desired sequence (minimum (50) and maximum (200) numbers of residues are specified above the window); (ii) enter the number of desired sequences (minimum (1) and maximum (10) values are specified above the window); (iii) select the target average disorder probability among values ranging from 0.55 to 0.95 with 0.05 intervals within preselected values. After clicking “Run InSiDDe”, results appear below the display banner “YOUR RESULTS” in the form of amino acid sequence(s) in FASTA format. The name of each generated sequence reflects the length of the sequence, its average IUPred disorder score, and the ranking of the sequence in that series. At the bottom of the page (not visible in Figure 8), a link to IUPred provides a way to visualize $\bar{Do}$ and ∆*D*o. The number of simultaneous queries has been limited to 10 to preserve the physical resources of the server. For the same reason, if an identical request is sent within 10 min, the same result will be returned without recomputing. Examples of IDP sequences generated by InSiDDe are provided in the Supplementary Figure S1.

### 2.2. Example of Use

**Figure 9:**
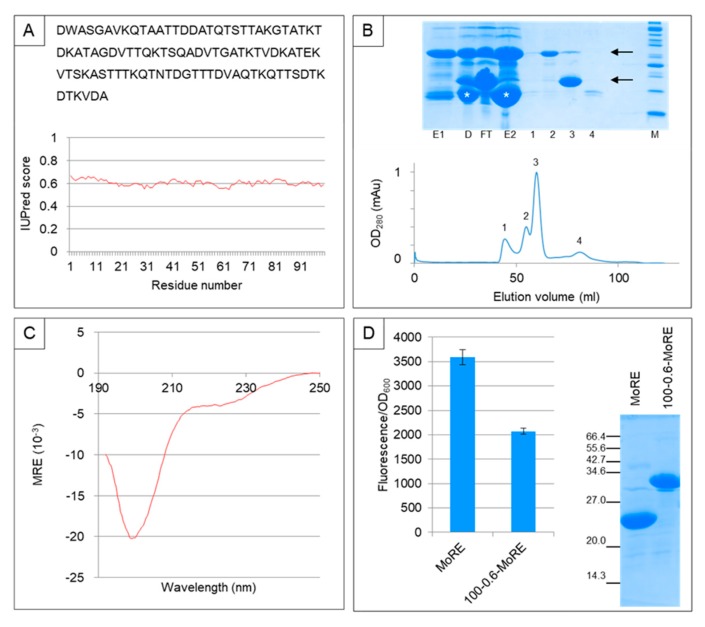
Expression, purification and functional characterization of a sequence generated by InSiDDe. (**A**) Sequence (upper panel) and IUPred score (lower panel) of the artificial sequence generated by InSiDDe with *D*t = 0.6 (100-0.6); (**B**) Expression of thioredoxin-6His-100-0.6-MoRE in *E. coli*. Upper panel: SDS-PAGE analysis of the result of the different purification steps. After elution from the first IMAC [E1], the fusion protein (upper arrow) was dialyzed in the presence of TEV protease [D]; The whole digestion mixture was then processed through a second IMAC. The second IMAC flow-through [FT] containing tag-free 100-0.6-MoRE (lower arrow) was further purified by SEC (lower panel). E2, elution from the second IMAC. The asterisk corresponds to the cleaved Thioredoxin-6His moiety. The proteins contained in the four SEC elution peaks (1, 2, 3, 4) were analyzed by SDS-PAGE (upper panel). M, molecular mass markers (from bottom: 10, 15, 20, 25, 30, 40 kDa); (**C**) Far-UV CD spectrum of 100-0.6-MoRE recovered from peak 3 of the SEC profile shown in B. The spectrum is the average of 6 acquisitions; (**D**) Split-GFP reassembly assay: Fluorescence values (left panel) and total expression of NGFP fusions (right panel).

Although the artificial IDP sequences generated by InSiDDe can be used for in silico applications only, they can also be used for recombinant expression in a heterologous host in view of various ensuing applications. In order to assess the latter possibility, an artificial sequence of 100 residues with $\bar{Do}$ = 0.6 (100-0.6) was generated using InSiDDe and expressed in *E. coli*. Its sequence and IUPred disorder score are shown in Figure 9A. Note that a W was added so that the amount of protein could be measured by UV absorption at 280 nm. A BLAST search in the non-redundant protein sequence database retrieved no sequence. To functionally test 100-0.6, we used the measles N_TAIL_-XD interacting system already used in our previous work [11]. To that end, 100-0.6 was appended to the *N*-terminal end of N_TAIL_ MoRE to yield a sequence referred to as 100-0.6-MoRE.

We tested the ability of 100-0.6-MoRE to be recombinantly expressed in, and purified from, *E. coli* as a thioredoxin-6His fusion protein. As can be seen in the upper panel of Figure 9B, the thioredoxin-6His-100-0.6-MoRE fusion was well expressed in the soluble fraction of the bacterial lysate and could be purified by immobilized metal affinity chromatography (IMAC). The thioredoxin-6His tag was efficiently removed by TEV protease digestion, yielding a pure non-degraded 100-0.6-MoRE protein of the expected molecular mass. Thus, InSiDDe is able to generate an artificial IDP sequence that can be efficiently expressed in *E. coli* and purified to yield a homogenous product. The availability of pure 100-0.6-MoRE allowed us to investigate its hydrodynamic properties by size exclusion chromatography (SEC) (Figure 9B, lower panel). The experimentally determined Stokes radius (*R*_S_^obs^ = 26.7 ± 2 Å) was larger than expected for a natively folded protein (*R_S_*^NF^ = 18.2 Å) [19] and much closer to the value expected for an IDP (*R*_S_^IDP^ = 28.8 Å) [20]. Finally, 100-0.6-MoRE exhibits a far-UV circular dichroism spectrum typical of an IDP with a pronounced negative peak at 200 nm and a low ellipticity between 190 and 195 nm (Figure 9C).

Taking advantage of the presence of the XD binding site in 100-0.6-MoRE, we compared the binding properties of the latter with that of the isolated MoRE towards the natural partner. We used a protein complementation assay based on split-GFP reassembly [21,22] that we already successfully used to investigate the binding properties of N_TAIL_ variants [11,23]. The 100-0.6-MoRE and MoRE were individually fused to the C-terminal end of the first two thirds of GFP (NGFP) to yield NGFP-100-0.6-MoRE and NGFP-MoRE, respectively. XD was fused to the N-terminal end of the remaining one-third of GFP (CGFP) to yield XD-CGFP. NGFP-100-0.6-MoRE and NGFP-MoRE were individually co-expressed with XD-CGFP in *E. coli* and their ability to interact with XD-CGFP was assessed by measuring the fluorescence of bacteria as described in [11]. In this assay, the interaction between the protein pairs under study brings the two GFP fragments in sufficiently close proximity to allow irreversible reconstitution of GFP. Hence, there is emission of a fluorescence that is proportional to the interaction strength of the pair of interacting partners under study [21,22]. Results definitely indicate that the presence of a fuzzy appendage made of an artificial sequence of $\bar{Do}$ = 0.6 generated by InSiDDe dampens the interaction of the MoRE with XD. This effect is not ascribable to possible differences in the expression levels of NGFP-100-0.6-MoRE and NGFP-MoRE fusion proteins (Figure 9D).

The agreement of the present results with those previously obtained using native and artificial truncated variants of N_TAIL_ [11], together with the SEC and far-UV CD data herein reported, advocate for the ability of InSiDDe to generate artificial sequences whose behavior is in line with expectations.

## 3. Materials and Methods

### 3.1. Set-Up of InSiDDE

The program was written with Python 3. IUPred was downloaded from the web server [12] under an academic license and used in the “long disorder” prediction mode.

### 3.2. Availability and Requirements of InSiDDE

InSiDDE home page: http://insidde.afmb.univ-mrs.fr/.

Operating systems: Platform independent.

Programming language: Python3.

Other requirements: Java 1.5.0 or higher, a web connection and a web browser.

License: This program makes use of IUPred predictions incoming from a public web server that is run in local mode. It is provided freely and “as it is” without a warranty of any kind, either expressed or implied.

Any restrictions to use by non-academics: None.

### 3.3. DNA Constructs

#### 3.3.1. Split-GFP Constructs

The XD-CGFP construct has already been described [11,23]. The sequence encoding the MoRE of N_TAIL_ alone (NGFP-MoRE), or the MoRE fused to the C-terminal end of an artificial sequence of 100 residues and of average disorder score of 0.6 generated by InSiDDe (100-0.6-MoRE), was fused downstream the sequence encoding the N-terminal half of the green fluorescent protein (NGFP-100-0.6-MoRE) by Gateway^®^ recombination technology (Invitrogen, Carlsbad, CA, USA) using NGFP expression plasmid pNGG [23].

The MoRE coding sequence was obtained by PCR amplification using Gateway attB-containing primers B1MoRE (ACAAGTTTGTACAAAAAAGCAGGCTCTCCGCAGGACAGTCGAAGGTCAGCTGACGCCCTGCTTAGGCTGCAA), B2MoRE (CCACTTTGTACAAGAAAGCTGGGTTTATTATTCCGAGATTCCTGCCATGGCTTGCAGCCTAAGCAGGGCGT), and the coding sequence of the measles virus N_TAIL_ as a template [24]. After DpnI treatment to remove parental DNA, the PCR product was used as the template in a second PCR amplification using attL1a and attL2a primers [11]. These primers contain attB1 and attB2 sequences at their respective 3′ end. They add about half of Gateway attL1 and attL2 recombination sites respectively at the 5′ and 3′ end of the attB-containing PCR product, allowing an LR reaction to be directly performed using a PCR product without the need of an intermediate BP reaction step [11]. The product of the second PCR amplification was used in an LR reaction with pNGG vector [23].

The 100-0.6-MoRE coding sequence was purchased from ThermoFisher Scientific as a synthetic gene optimized for expression in *E. coli* and flanked with attB1 and attB2 sequences. After dissolution in 50 µL of 5 mM Tris/HCl pH 8, the synthetic gene was used in a PCR amplification experiment using attL1a and attL2a primers. After DpnI treatment, the PCR product was processed as described above. A detailed description of the two constructs can be found in Supplementary Figure S2.

#### 3.3.2. Thioredoxin-His-Tev-100-0.6-MoRE Construct

100-0.6-MoRE was PCR amplified using 100-0.6-MoRE in pNGG as a template and primers attB1-tev-106 (ACAAGTTTGTACAAAAAAGCAGGCTCTGAAAACCTGTACTTCCAGGGTGATTGGGCGAGCGGCGCGGTG) and attB2 (CCACTTTGTACAAGAAAGCTGGGT). After DpnI treatment, the PCR product was used in a second PCR amplification using attL1a and attL2a primers. The second PCR product was processed in a LR reaction as above using the pETG-20A Gateway plasmid. Primer attB1-tev-106 encodes a *Tobacco etch virus* (TEV) protease cleavage site immediately downstream of the attB1 site. As a result, cleaving the thioredoxin-6His tag leaves a non-native glycine residue at the N-terminal end of 100-0.6-MoRE.

Constructs were all checked by DNA sequencing.

### 3.4. Split-GFP Reassembly Assay and Purification of NGFP Fusions

The split-GFP reassembly assay has already been described [23]. NGFP-MoRE or NGFP-100-0.6-MoRE were individually co-expressed in *E. coli* with the XD-CGFP (the binding partner XD fused to the C-terminal half of GFP (CGFP)) and fluorescence measurements were carried out as described in [11].

Expression levels of NGFP fusion proteins were assessed as follows. Transformed cells were those used in split-GFP experiments, except that only NGFP constructs were expressed (i.e., only isopropyl β-d-thiogalactopyranoside (IPTG) was added). For each construct, a triplicate 4 mL culture of terrific broth (TB) (DIFCO) containing 100 µg/mL ampicillin in a 24-wells deep-well was seeded with 300 µL of an overnight pre-culture grown in LB containing 100 µg/mL ampicillin. The culture was grown at 37 °C until it reached an optical density at 600 nm (OD_600_) of about 0.5. IPTG was then added to a final concentration of 0.5 mM. Protein expression was allowed to proceed for an additional 3 h under shaking at 37 °C. The OD_600_ of each well was measured, and a volume corresponding to the same total OD_600_ for each construct was spun for 5′ at 4000× *g* in a single well of a 24-wells deep-well at 10 °C. Cell pellets were re-suspended in 1 mL of lysis buffer (50 mM Tris/HCl pH 8, 0.3 M NaCl, 10 mM imidazole, 0.1% Triton X100, 5 mM phenylmethylsulfonyl fluoride (PMSF) and 0.25 mg/mL Lysozyme) and frozen. After thawing, cell lysates were supplemented with 20 µg/mL DNAse I and 20 mM MgSO_4_, and incubated at 37 °C for 30′ under shaking. Urea (1 g per well) was added and allowed to dissolve and denature the proteins contained in the lysate by an additional 30 min incubation at 37 °C under shaking. After spinning for 5′ at 4000× *g*, supernatants were supplemented with 50 µL of a 50% suspension of IMAC sepharose high performance beads (GE Healthcare, Buc, France). His-tagged NGFP fusions were allowed to bind to the beads for 30 min at room temperature on a rotating wheel. After washing 5 times with 1 mL of 50 mM Tris/HCl pH 8, 0.3 M NaCl, and 50 mM imidazole, beads were re-suspended in 50 µL of reducing SDS-PAGE loading buffer and the proteins contained in 10 µL of this suspension were resolved by SDS-PAGE.

### 3.5. Expression and Purification of Thioredoxin-His-Tev-100-0.6-MoRE

*E. coli* T7pRos cells were transformed with 100-0.6-MoRE-pETG-20A construct and incubated overnight on agar plates containing 100 µg/mL ampicillin and 34 µg/mL chloramphenicol (AC). The next day, cells were scraped off the plate to seed 1 L of TB-AC and were grown at 37 °C under shaking. When the OD_600_ reached 0.5 to 0.8, IPTG was added to a final concentration of 0.5 mM, and cells were grown at 24 °C over-night under shaking. They were then collected by centrifugation at 4000× *g* for 20 min. Cell pellets were resuspended in 100 mL per liter of culture of lysis buffer supplemented with a SigmaFAST protease inhibitor cocktail tablet, then frozen at −20 °C. After thawing, cells were incubated in the presence of 20 µg/mL DNAse I and 20 mM MgSO_4_ for 10 min at 4 °C on a rotating wheel, then disrupted by sonication (3 cycles of 30 s each at 50% power output of a 750 W sonicator). Following the removal of cell debris by centrifugation at 12,000× *g* for 45 min at 6 °C, His-tagged proteins contained in the supernatant were purified by IMAC on the bench by gravity flow using a 5 mL His-Trap HP column (GE Healthcare) previously equilibrated in buffer A (50 mM Tris/HCl pH 8, 1 M NaCl, 10 mM Imidazole, 1 mM PMSF). After washing with 25 mL of Buffer A and then with 20 mL of buffer B (buffer A with 20 mM imidazole), the bound proteins were eluted with 10 mL of buffer C (buffer A with 500 mM imidazole). His-tagged TEV protease (1 mg/20 mg of target protein) was added to the pooled elution fractions containing the protein of interest and the whole mixture was dialyzed overnight against 10 mM Tris/HCl pH 8 and 150 mM NaCl at 4 °C (with 50 mL of dialysis buffer per mg of protein to be dialyzed). Dialyzed proteins were loaded on Ni beads as above and the flow-through containing tag-free and Tev protease-free proteins was reduced down to 1 mL by concentration using a 10 kDa cut-off Centricon (Merck-Millipore, Guyancourt, France). The 100-0.6-MoRE contained in the non-retained fraction (flow-through) was further purified and analyzed by size exclusion chromatography (SEC) using a Superdex 75 16/60 column (GE Healthcare) and dialysis buffer.

### 3.6. Hydrodynamic Analysis

The hydrodynamic radius (Stokes radius, *R_S_*) of 100-0.6-MoRE was estimated by analytical SEC. Typically, 0.5 mL of a 5 mg/mL solution of purified protein was injected. The experimental Stokes radius (*R_S_*^obs^) was deduced from a calibration curve obtained using globular proteins of known molecular mass (MM, in Daltons) and whose *R*_S_ (in A°) were calculated according to [25]:

log (*R_S_*^obs^) = 0.369 × (log MM) − 0.254 (1)

The *R_S_* of a natively folded protein (*Rs*^NF^) with a molecular mass MM (in Daltons) were calculated according to [19]:

log (*R_S_*^NF^) = 0.357 × (log MM) − 0.204 (2)

The *R_S_* of an IDP (*R_S_*^IDP^) with *N* residues were also calculated according to [20], using the simple power-law model:

*R_S_*^IDP^ = R_0_*N*^ν^ (3)

Where R_0_ = 2.49 and ν = 0.509.

### 3.7. Far-UV Circular Dichroism (CD) Measurement

Far-UV CD spectra were measured using a Jasco 810 dichrograph, flushed with N_2_ and equipped with a Peltier thermoregulation system. One-mm thick quartz cuvettes were used. Protein concentration was 0.075 mg/mL. Far-UV CD spectra were measured between 192 and 260 nm, in 10 mM sodium phosphate pH 8 at 20 °C. The scanning speed was 20 nm/min, with data pitch of 0.2 nm. The buffer spectrum was subtracted from protein spectra, and the result was smoothed using the ‘‘means-movement’’ smoothing procedure implemented in the Spectra Manager package.

Mean molar ellipticity values per residue (*MRE*) were calculated as

(4) $$\left[MRE\right]=\frac{3300m\Delta A}{lcn}$$

where *l* is the path length in cm, *n* is the number of residues, *m* is the molecular mass in Daltons, and *c* is the concentration of the protein in mg/mL. The number of residues is 123 and the molecular mass is 12,620 Da.

## 4. Conclusions

Although we illustrate an application of InSiDDe pertaining to the modulation of the interaction strength between an IDP and a structured protein, we would like to emphasize that InSiDDe can be used for more general purposes, such as the fast generation of disordered sequences for further in silico analyses such as those reported by Nygaard et al. [15]. Future development of InSiDDe will involve the possibility of designing artificial IDP sequences fulfilling additional constraints, such as specific amino acid contents (proline, glycine …), hydropathy, net charge per residue, and charge distribution, to mention a few.

## Supplementary Materials

Supplementary materials can be found at www.mdpi.com/1422-0067/19/1/91/s1.
