# Supplementary material for: InSiDDe: A Server for Designing Artificial Disordered Proteins
Source: Int J Mol Sci. 2017 Dec 29;19(1):91. doi: 10.3390/ijms19010091 (PMC5796041; doi:10.3390/ijms19010091)
Supplement: Supplementary file 1 [file ijms-19-00091-s001.pdf]

Supplementary Figure S1

Dt # 0.55
Seq_1

TYRDDDRADIDARIRTTDRKTRKKSTVSKIKKLGTKGIDEDVTADDRDTDTKVDADRTDKRDYSRTEKATTKERDIV
DETIVSKEDDEKKRKKDEDQKTT

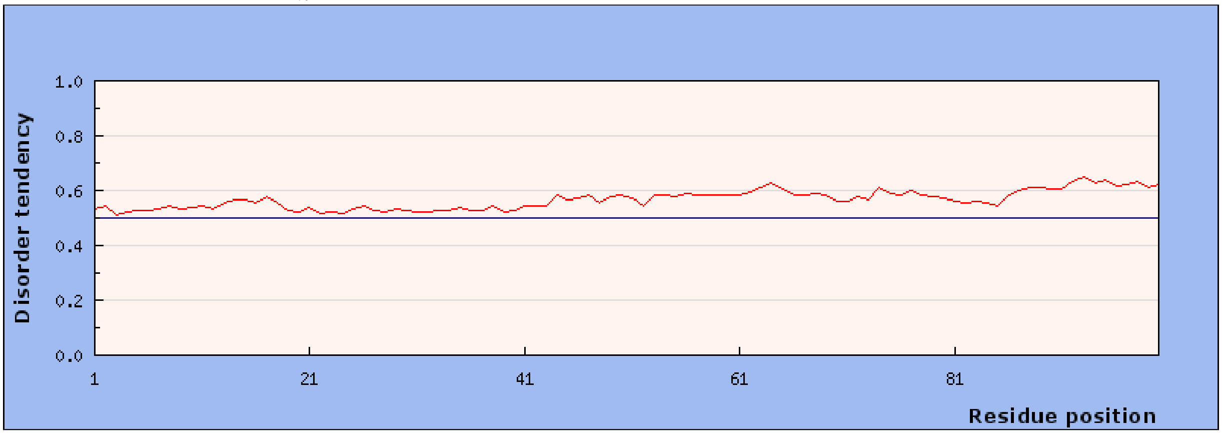

Seq_2

SKDTTTYQDTDTVTDRDDYTDKRDTRETTKDDRGTGDTRKKDTKDHDTTSVDKTKTRGSDDDSTKSTKDKKHDDDAV
DTDTQDTKDDRAKTRDDKDDKRG

Seq_3

TKALETDTYQDDMKGLSDMDDTTYQLTTKKETRTKDDTHDDERSYTLGDDKGKGKVTRTYDGLKKRQDFETEGAKTD
KRDKKSTTITGKGDRTKDKKGTT

Seq_4

RDAEDKRIARRESRTLYDEQKSEIDDTRFKTKKEQYGKRETREDLVREKRRTQYDRKVQTERIGQDDLRYRQTEEDG
YRRKTDRKVYYDTKDEDDGKVKV

Seq_5

REMRNRKRRIRQVTRGDIYDKRETRGKISDYTRTVREYRGTVSTRGRIEERYQKGEDYRYEARNRYDRSERYDREQV
DRYSERKRDREYKEENMEKEEIR

Seq_6

KDTTSDMTKSMDLNKTMKHTTTGLMVKTDHKTTTSSKMDTTHTDSTYVDRSTRYEKMKMTLSKDTKDDHVDSTTKKD
TNDTKSMDTTGTDSKDLTMMTHK

Seq_7

SSKISEKTTTISGGAEVYRDKYESKGTTKAKKKSEEGEYKMDGIKTTQAKVGGTMKTGDGSTRIKNYYEEISKGDYE
NKESTRYTKTDTKAKGKLSKTTG

Seq_8

TSMKTKDKDDKSMHDLKGDTDKTTGHDTKTDNMTLKGDMGKGDLDDRVRYETYTSDHDTKKITDGKGTTDDKSVTMN
DTLNTMTDTKMTTTHGLDKTHMD

Seq_9

NEARRDRRIGDYGDKMEERNRKDEYREAKRGIDYDDRSTRLDKQRKRARDDEDIKTKEDYEYTVYKESRRIDKDDSI
RDGIREKRRRIESSDEYKGERYR

Seq_10

KGNLTTHMDMGKTKGTDKKAKGDDMYRTEVVTTGKGDMNDTKTKKTDDYQTDKRTATVGGTKTTLTKAGKDRSYTTL
NTTISGGTLGDKDTKKTSDLTGS

Dt # 0.6

Seq_1

SVEKQTQELEKKDIKRLQKEQKKSTKSSTVEKKTKSEKKVDKKSKSQTEQETVKKQTKEIETSQKQSVSEKEEKQEE
VSKTQTEKQSKETTRKKQKKTSE

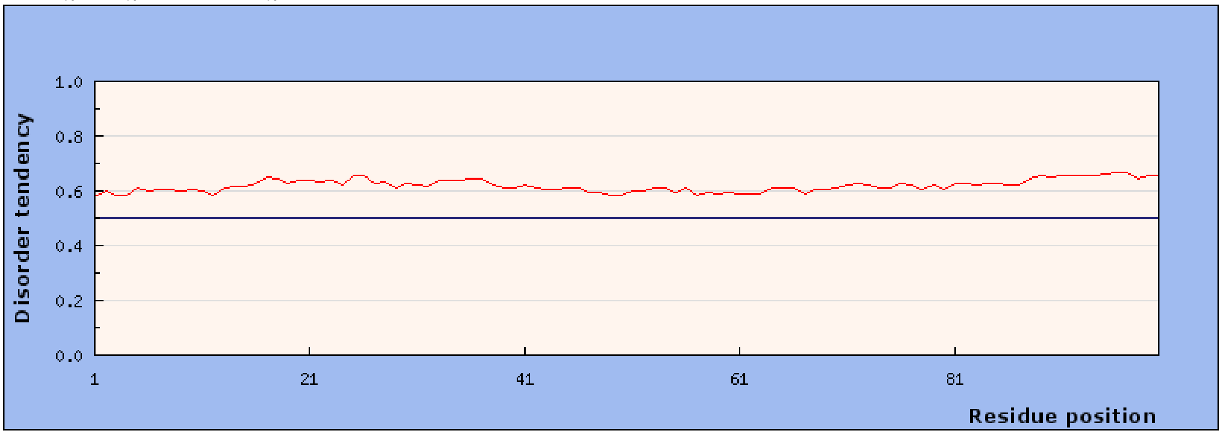

Seq_2

LSTDKDKEEAYSKDSAETEKSASETEYRSKEESKKAKKKDGKKTTSKKDRTYESDDKASVKTSVEATVTKKDSDTLS
KKKKDSKKTEKDRTKKSKKKEES

Seq_3

NDGTLKTSTVTDKKTDDNTMTHSTKMASVTNGMKTMTGLDDRTTDVTNKDKTMSTTIHGTNTGKDDDLGTTKDHTMT
AVDTTLGHGQDIDMTKDNDHTTM

Seq_4

DGTLGTTDDHHDATMSNMADTDSMVMAVHTNDTMKGKGTDKADTMDNGLKDTGMTGGTDKMTDAKGTLDGTTDKSTT
AKHTATTKTSVTKTTRIGMNDKT

Seq_5

VDTDRKKKAGTDRSVTSTTDTDTTDTLSRRTDTVTTTIGTLDSTTSRTRDTDDVTRTSRTDDTRHRDSDRTTSTDRV
DSRVRDDRRTTDDDTTSVTSRTD

Seq_6

VEEQKSQSKKKTSKKTRYKDTETAKQSEKKQKTDEQSETVEKTSKKKTKTAEVESEVSQKEKKQTKIKSKQKQTEVE
SKKKSQLELESDETKEVESTQTK

Seq_7

RTVDDRARDDKRLQDKKLGSKKKDTKGDVKDRKIMTTLGETKKRDRYDVTEDRKQMIKEDRTKKDKKDETTDKREVE
SAKTADKGYKTESSIDTTEQRYK

Seq_8

EKQSQVKKSKQKSKQEQKVTEQTQKDEKKKVKKETKKESKETQVTQSVETKSQTTQSKEKQQTQEQSKESIQKSTTE
KVSQSKETTEEQKTKEADKQKTE

Seq_9

QQMKGTGLKANTTQTVTQSLTQSVTGQTKMTQNDTDSKKLMVQTTTQSLTTNTDVGQTKGAKGGTLSVTTQTGVTTT
DNLTTDTNSTDVLSKKDGGTTVT

Seq_10

TLRDTTRTTSRDRVTRDLSSNRKVESRDTRIRRDTTADTRTTDRDYTYDTNRTGDDTSRVRDDSIDTADSITDDAKD
DYDTRDRTTTSDDDVDRDRDRSS

Dt # 0.65

Seq_1

KQTGGGTVAATQSAGTGGKDTKEEDEYRQTLKEGKNYRDKGDVKKDKQVKTKGTGTKQTGKVDKTTTGKQTQDKKIS
QKKVTQKKVTKKQKDAREKKVTD

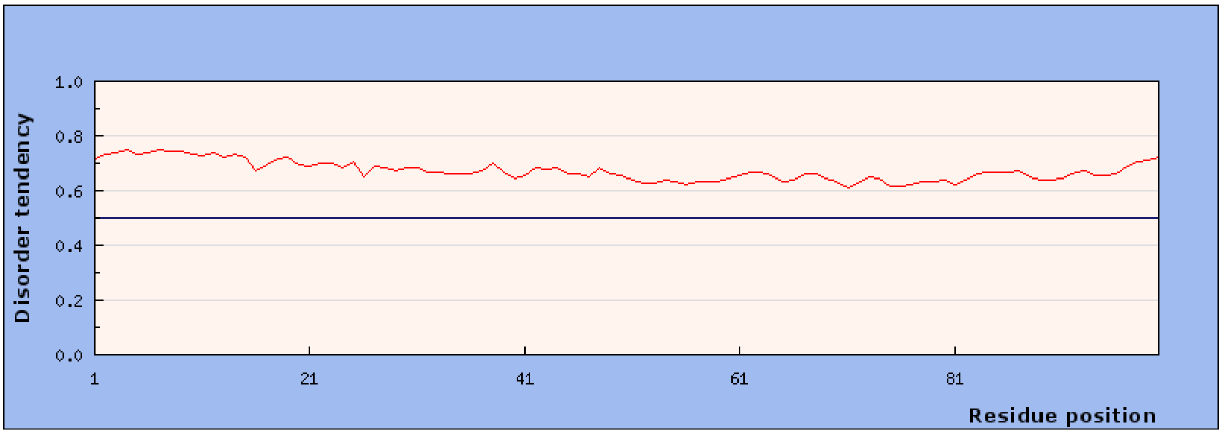

Seq_2

GGAATDGTATSVGSTVTRADAATQKADTQTGRTYNSMLDKSTDTNSKGTTVTNKASKMSIGTGNTGDKKDAKTAGGT
DTAKGTDATKASDGSKAADTKSA
Seq_3

ATSVTSKAADSSSMDDTTNGRVTKDVKTQTGTKDQGVDATNISKSGVTSTKTNAKTTSMDTARKDAATATGGKKDAT
DGAYDKGHTNKITTTAGGKNKTG
Seq_4

DLSKSLASKEGKQTEGTNSTLTETTSSGKDTKNTAKTDYRSDEKKYKNDDKTKVRKQIETNDKKVKVGSTSDKLTKT
RHDKVKTALKKTGQTSTKSKTDG
Seq_5

QDDTTMVDRTTRDDSDEYTQRKVDKKRRNGRTTELDTKRQKDTNKGTYEKRKQETYVDKRTEQYKKTHTDDDTTDKD
YNTKDRRTTTMLGEKSVDEYKRR
Seq_6

NTDGGDDSTSTLKGDGDDTGTTKTKTKVKNRRDVTTKSAAKVRTDSKSVADTTGSDYKKNGDTGAADTATATSGTAG
VNSTDGKLDGTDGKLSGKTMLSK
Seq_7

ATTTRVRKDTVTDGAYDKRTKTKDENLTSKEMRKRDTRLDADGVAVDKNKKRTDYAETRKTGTMSVDDDGKGDVEHD
GKDKDKVNADRKTDKYKNVADRD
Seq_8

NMGKKITKTDGDGTTTSKDNETQKKTDLDGIQTMKTGKNIKTRNDTKGGDTGDDLDGYGKDQTTMNGTDTDGTGKLT
GKGEVGLKLDDKGNGQMTTTVGG
Seq_9

MNIDQTKTEATAKQAQVQTGSVKTNTRDDVYKKTREKGTGTVTNDTNDKKDRYVKTSSAKKTQTVDDKNVYNKKKAS
TDTKKSTAMTSTQSQLSKKGTTT
Seq_10

QTKDYYERDTLSEEKESLNKSKESKVTQGDKKTEGKIDEQYKKKDKKQTDEQRYTTRNVDEKKSTQKGLTKGKKTAE
LKTVTKDGAKSKTTKQQKRQTKE

Dt # 0.7
Seq_1

TNSEQKTTKSKKTNSQKTEQTNKGQQSKKQKKTTVNTEGKKKVTQIQQRTQKEDKESTKNTQTTIENQSKQNTEQET
TTIKSQNQTEKVQQKNEVQQKQK

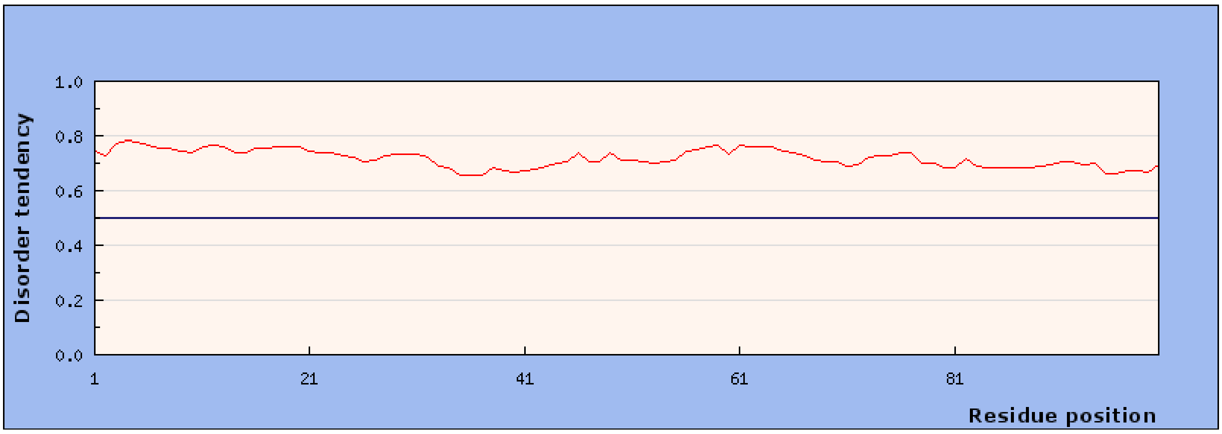

Seq_2

SSDKTVRTKVERSDKKDEKANKTTLKAETLGDLNKSRETKKKKEEERDIRDGTDTGQSISVTRVSTERTVSKNTTST
AKESREITASTLSRTSESSERAL

Seq_3

STVSSNAGYTSMKKTTNNKGTTQKGDTSTQKTDYRSQKTYHGDVTGKNTKDNTISTGGVTTQRDKTKQQSYDKSRET
NITDSITKDSDTGDDKSRKTTHM

Seq_4

TGAQSAQQQGTITGQAAGATNGTTGAQAQTNKLAEAAAQSGVNSTRTSGYDNVSSSTVGGTGQAQTTSKTAQGASVS
VSGQQSAQNTTEKSAAATQAAAQ

Seq_5

GGNDKGAKDKGNKTMKKTSTTDNGDKYNGTTENKRDDGMGNGIKDGNIKDSTKTNTNSTKKGKANADTDYRASDDAG
MGKNDGNIDGDGAGMDNGDAMGT

Seq_6

YTNKDEMERARREGMYAKGDRDGKYETADEERNTYDESNTYSGTRRKDGRYRTSDKDRRDIKRTKTKDADIKKRSRT
DRSRNSDTKDNEIDAKRMDERAT

Seq_7

DKTVRYDTNADESTTGNVSVRKTNKSRVETSRKTSDTKRIEERKLKEKIETRSADGQDKTEEAKSQDTDYTVRKSTI
ESDGTSGTKKNRITEDKRETLNG

Seq_8

QREQTKEAVQVSRTVDIRKEDRQEKIDRRTTRQERGQQEDQKRQKTRTTQIERDKETIDQQLKTKQRYQTEKTTQTQ
IKQKQQTQSRTKEGERRDTRYRQ

Seq_9

TVTANKKNQTTKTEADSRTYNTKQKQTQVTQQSKKQQDQGTHGDTSTTLEKKANTVTGVGETLKKDTRYTATNTNTS
EKVQKKTQTLKSNEVTKSTQTNQ

Seq_10

ETSLDTKQRESKRDDKEAKSLETKASDDGYDRGYKRGRRYKDTRSKRTSDRYRDGTQDYSDDTKSTRRSDRRRTKSN
TKTIRTEDSYQTTTRTIDRSAKS

Dt # 0.75

Seq_1

MDEGTADGVTGKNSAETMTEARKNGKGDARAGDVGDRTVGDKDDDSKHDDIGSTKSHRVKKGADGDTRKGNKDDVVD
DDKDKVRRVKETTKGRGDKKEAK

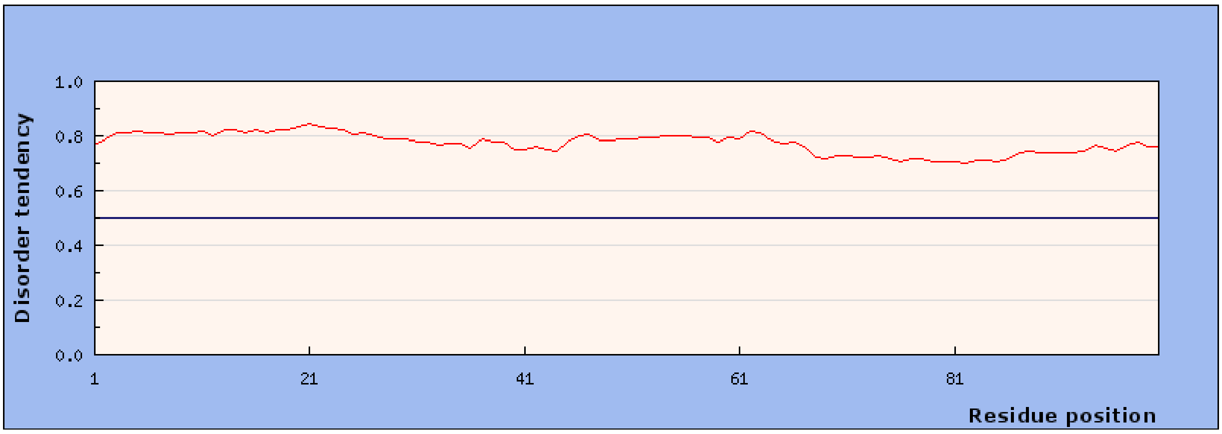

Seq_2

KSAAGGNTADTELTGGKERTTSDQLTVKNKTETKTNMDTTNDMDRKKDLSREENGSTTSISDSIRKGLRMASETGRT
GSTANYDSTKKMRYTKNQNSSKT
Seq_3

EGTKTSQDKKKDAVEEEKSTRTEYTTNKGYRVREKDSVRSTVEGKTQVDERQGTKRDGKMEYDKDTTDEMQEEQKVK
EENRKELNKKDSQADVKEDVQRQ
Seq_4

EGGMDRSKDYGDEYKRQDKKKEKNDKNDGEKRMEDGDKREERTKYKEEESKRNNVEVNTEGMDTKVRETTRSAYTRS
TVTRDLDSNRTASVEDDSKRTKD
Seq_5

EVSSRYDDEKAKDEYEDRNSKADDKDDGKERKTLMEESKRDRDDYKQREEVTSRSRNGNYDNGLKKGTTESSKVRVR
DNGKLEKGSRGTNRSVSDDKKGD
Seq_6

DTTSNNTMRTDERTKMVEKGRDMDTNYRYDTDESTKRSDDVTRDKNQYNDTGTAKNSKETDHKDVVNRKKNGRDDVG
TTNDTKGRTSVKNTDGDVDMSIR
Seq_7

ETKVERISTKRTKETAGKVDSDYRDDSERAQTKDGVRTDDVANDGDADKQSKKEVDDKKRKNMETQTREVSYRDTAN
RRTGTGRDGMTKLETQRDMTDRR
Seq_8

RVDGENDKKVSTVNGDSSMKKRDGVDTNDKQNYKTNNYAGGTDTMRMGGTKEKDVDVRTNKQTTDTGADNRTEVMKD
ADNAGSAKKTAEKDDGARTKKGM
Seq_9

MRVGELQDTREKSNGNDERTDRKDGKTEVEGGTDGKVMKGKNVDERTVKETEMDRKVNEDRGEDTRDVAKRMDTRAD
VNKVDMTKKEKAVGTRTAGDKTK
Seq_10

RSKVKNGDDYKTSDITGDVRQDKTTRDDEKDNDVEDTNDMERKKSETGKNKSVKDTNLEERSSTIVKEKNTDARSEI
RSRGKDAGEQRTRDYRRNDDHDD

Dt # 0.8
Seq_1

TTSEATNYQESEKQGKVRRDQQEKREDKQYKRRSAQRKDDSKTASEDRQESYKQQRKDKVRVKGDEEKTSREQQSNY
TTGDDKQGSSKEIQKGRTRRVDQ

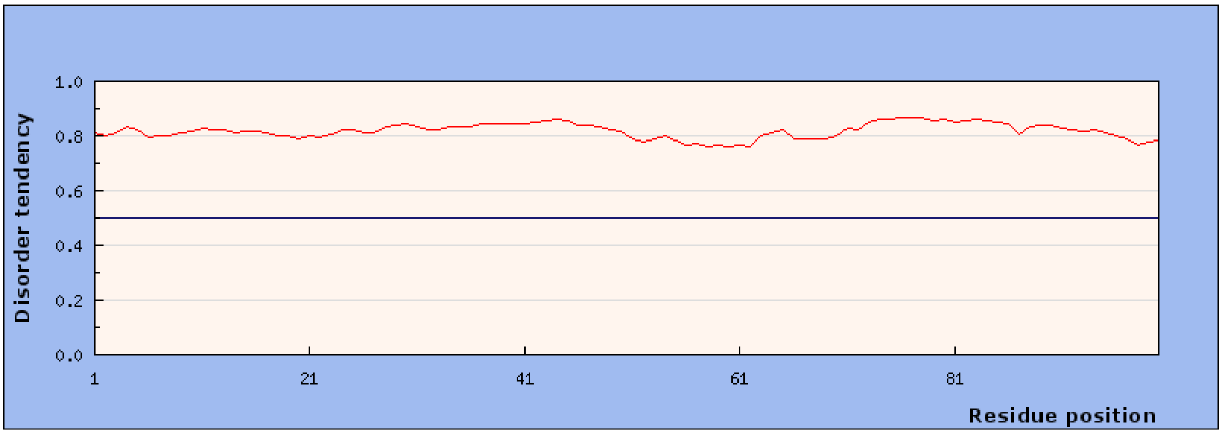

Seq_2

RGKTASGGTTNMKSTQQETAGDKRTATKGASAGTDGSTSGMGATKTNSDDKSDGLSKSNSGGKKNNTGDTDAQDKTK
NAEKNYETSKSGADADTNDETNK
Seq_3

SKEVQDRTKKGKRSSKSTKDNERKETREYTTVDYNTKSRADDSGVVAERDTTRSQNNSKVKESERNRKSDGNQQKES
VGSVSARKEEENVSKVSAEEGNQ
Seq_4

RIMETSRTQVNENGNKQRITGLEKNDSVKKSKTDNQRTTDDVRGSSVDVDNGSKNDASMKIAEKDLDDGSKSRTARR
ENNDEDTARRELKAERTDGGRDT
Seq_5

IQEGKTRDIEERGVSNGTDGNTNDTDYSNYKNQYADKDNNQERKDDYTNNSNYGRDAITTRYVTSETETQTRNNGTR
AAVGKLQSKNEESQDAKTKSSES
Seq_6

EENSVQGGSREIESSNNGEKKNDSRQDQSYRARKKRSTNKGRSSERKATDSSVKKVNEKEEYESEESESTEQERKER
AVSRERNLKQGSKSSVASESGRK
Seq_7

DQSRVTDLEKANADVQSEEIGQDKRNYVTSMNRLKDNTNNSREAKDEMKDRGRVSRNVDSYGRYSEDGTSKDNDRRD
VDKKGSKSQRDRRHSGNVETATR
Seq_8

EDKEYESAKRGKSSQNSESRKVRDKSGKISKEEDQKSEISSQEGSSHGLTNKDVEKSKIESGKEQSQYQRSEKQEDR
GQDGVEEKIKSEENVSDRQQQRS
Seq_9

NTMDGGKSMAEADLNDANGEKSAMKKEEGNREMRGVNATRRKADGDAVSGRMDSKDSSMVNTADTGDTITLNRRNGS
SNTRLKTKRKGTKNSKSTVDGGS
Seq_10

DKGKRSATRSVKDQDQSKTADDDGRTGSNEDLERKEKGDSATKDRGNGKKLTSEDDIETQNTLGKTNGKALDHVKRK
KVQSTESQDGRKRKRKEEDVDQR

Dt # 0.85
Seq_1

KMGRDEQSYAEKYGEMRPRVEKLNKRDKNEIKEKDRYNDSSKEQRETSVSTTSQDRYGGDTEHDADRHTPYNQYRGS
GKRARGSEDKSEHRNKDTDAGNN

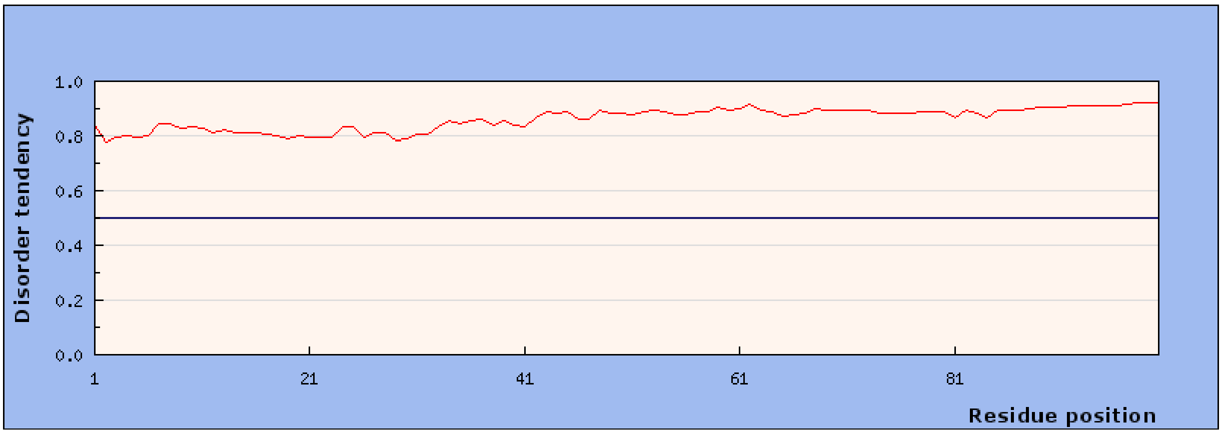

Seq_2

NDTRTSESQERNTEEDRETEEERANEQEDEGNKSETRLKGTGNTIFTEDDENGRNEGEKTNKRETDKLGERKNSTDV
TKTKTQNQQKQTDKTQRDNRRQD
Seq_3

KGTERIDQETPSARRSREDYDSNEVEQDNEKVESMGGTIKHSSKTGKQSLKKDARGDAGVTEETNKSNDDTKTQRMR
NRDQGRGKMRVGENSRSKRNLTK
Seq_4

ESTKRVSMARMGNQTVSNSPKVDNEDYNSADNMDSKGYESLEENKYNDEQYKNRRASNTKDHEKEEVKKSTERHDKD
SMSMTADTTVQSDEEDKEGSDNI
Seq_5

QEKNENKRTKNDNTTERDNDEENFDTTNEGRDQEKKKSGGDEMKSSDVTNTSPMASNVDKRLEKNNSTSIKLRKRRT
GNDTFGENRRRQDTRGELREDST
Seq_6

ESTVTETNQASEADKLSNTNNADEKRSYETSLNKKEVEGGMGNKVVDSGTSGHAKSGEESKNSSMKEGRGTSPSRRR
GGDGNSESTYQRNSKYRDDSQST
Seq_7

EADYKRNYDNADRDGEGESDREESKYRQSEERNQRDEEHYGGKSSTDKRKGESNGNEVMEEGKKSQGSQEDGKRGRN
IPAHKYKEENRGKQEKYRSNTQR
Seq_8

AVTTEMESSKNEKGRTELNSDYKQEKKGRERDRDTDEVEMKLDGRGNKLGDEKESQSKAKKARSENRQTNNSLGKAR
TSNNKKEAGADDRSPMTAEKDRN
Seq_9

KSETRRQQKDNKQTQRNQTTQKTRSRQNRERKNTETKDVTESYTSSDTKGGKRENRSRTPAEENRGRLTKEEYSNES
DQEDDRNRTERDEESTESEQQSD
Seq_10

KRNRDESEEDSGRAVGKDTNANKNLTNERTVKMSERADEGETLNTDGSINERSTKAGRDTEAKMTADREEATLDGKS
ETEAGTNRREPMRARVKMGEGDA

Dt # 0.9
Seq_1

MPANTKRLMDDTRTSRIGKMNAKNKRTGAIETMREMKPDAQQKMEMQGRNAEKMKLDNDRKTTNNQNGAMTPKKKTD
PTMDKQRTMNRSGEQDQNDNTKK

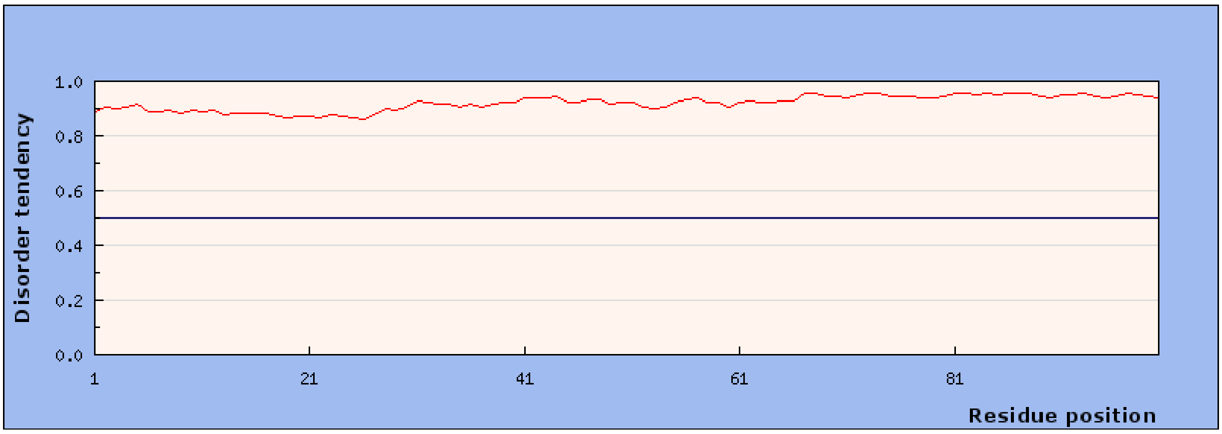

Seq_2

VDPPANIPKGPHSRNPGRPSISKSMPAPDHPSIGIKKSSMHPAPVKIENPMGSSKPVDRPIPGENQRHPNPVVRPGP
NKENIIQHIIPQGSKSMEHPPVR
Seq_3

RRMSDHKSQSIRHSMEDQDIDGDETNDQRLESMHAQTYSPRSTEVAGENLHNTSTKHDNKSNGRSRNIQGMKDEQRL
GHEARHKDAENQGYERSDSGTTN
Seq_4

DQARDNKDANSYTSQGDELEHTDSATPTKNSKTAREMKLGGRNKNKQRVNSMTSENTQYGERDQHGAHDTKADNMKR
TAGDTGRHSPGNRKMTPTGKRTK
Seq_5

DMQHSNKQDSTSVRAQGGEKNMAHGNTNSADSVQDHRTTAMTSDNDQKKSQSMVSQTVNHGAKSDSNKMKGHTLENT
PLKPKVGSDQDEMAKSKDTNVNT
Seq_6

EGTDRGQKRIKNNERMMRANESQISRMTHDDNRRKTMEEAQRENMDKAGRQRDKTAAEKRQNGMRAISRNDQHEITQ
RRMEEGQTGEDGMKRDMTDHEGL
Seq_7

NKNARERHMDMEDVRQDKNNKGLELVSKKQSNDKKDNHQTSYGQQNRVGSNKQAGDNNKKTSNMVQSVMDEGPGGQT
NPNKYGQSRDSQNHQTIDVQSGG
Seq_8

GTSNEQNLLPTTKARHNATLKTNRVHTMEHETASPKRTSNMKSENNGQNKGNTRYSPGKIVHKEDGIRTRDQIRDHD
SKEGSNETHDGASEKTGNLNTHA
Seq_9

KSKKSTTGKATQSVNTGKKRMSNKIGMASIEMRKKNDEQDKSNGKSDQVSNKKDMMEPSIQRLMKANQGMQEHNRPG
KSEKATPIHKMTEKRPIKAQDND
Seq_10

RQETDLQNEESTEVHIDADHAKTITDHRETKRDSKQMAMERSEQMSMQIRESTQRSITQRREGNTNVHTELEGGDRK
GPKEKDRQNMTQKMNMKPMHIHD

Dt # 0.95
Seq_1

PKDMGEPARSPHVTRNESQRRTRVYTAAGLEGMNALVNKDQHGSRKQKNTGEMPMDMRVRNPHDEPNDQMKHRHKNQ
PHNTNRSNNNRYQMIDQTDHSTS

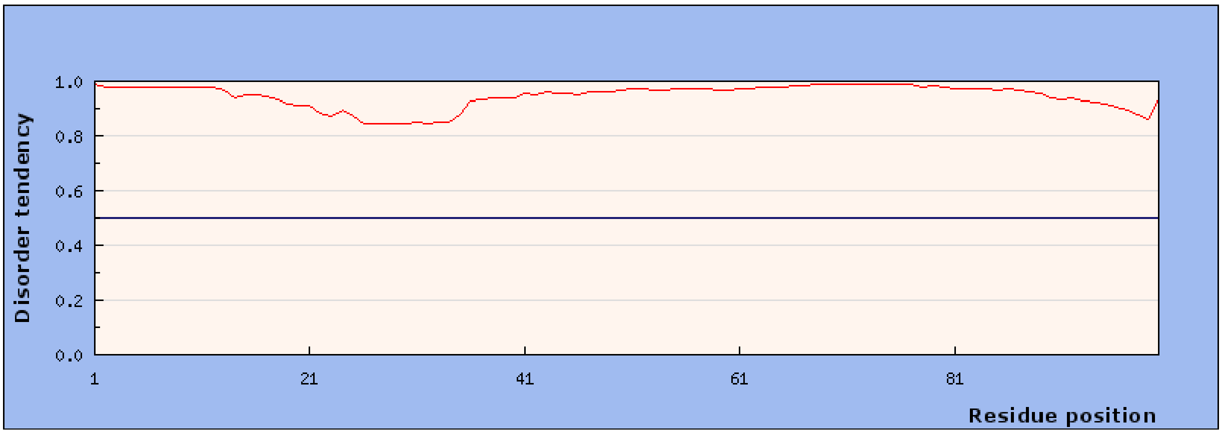

Seq_2

RMIGHKVTAHKDNINKRTGGDRPGNTAGGHRDLETSEQDQRGAEHYPRETPQQPLDPEAVHMTGKPRNQKDDLSESG
LGRHRRLMQPESAQSRARPEKGN
Seq_3

QDTEKDKLNMMPKMKRNKRHGRPDKQPMHLHVREDRRVKTSGDQTNENRQSRNSTMRPTIHSQAAAGAEPRDAEDDT
SGVDRVNYQTGENEDNHQNLRRP
Seq_4

RPLQKRRYDNESTNVQHSQKNRDLTNSLESTSRNNSENIKGLSDDGGPRVKSDTDDTGSEPANDQRNEQSHMQTEER
PKQTDKKSVDTPNNVKHRDRNTE
Seq_5

DDKSTMRIDPRLQDMSTVDEPRKTVSTKGRKHMRTPDNAKYSLESKQESPNNADIPHQDEFNQHAGGRLREPPLTQR
TLHNVTNPDAHEPTAQSEGMPHS
Seq_6

AHEGGHATDGGQMQTDQPQLPKPMIVTRPSQREPTVTHRLASGDADDHNKNLEVPGKEHNRPKTQMASDIGDKDGKQ
PEDHVTGEHDSKEDLSEGSDAKN
Seq_7

PEDLMPVHSAHNDDNASHVGAMRQHKEQNLVKDPLVQRRGPAAHDSRRTKAAQVHVEERGNRKDKDNHNENHDMDSD
TTHDKRNELMKEQGDQSMQDARH
Seq_8

HPLNKKMTEGLKQRPSVNDTTEPLGDRETLPDSSATYQESQELSMKLNTRDREQPENTHSPNDEVMHQGVQPRNKQH
DQVASSPQTTHRVKNQPMDVEDN
Seq_9

SNPATKANADKGENKHSYELQRIPTVTKGGPLSGRTRDDSTKHTYAMRPGPQSLEHSHHNMRPNGNGRGDPNHDAAM
DSEQPDSEQTPMMSGEHGHSTSP
Seq_10

PQEHDRPTSMMKPIKDKSEQNTHHYHKKGLVSTDGRDRSMPSTATRMDPGNRDTSSHPSNVRQGHRPDGKTHAARTK
HHSQTMGHQPEDPDGRDNGLVQT

Supplementary Figure S2

NGFP-MoRE

```
--------T7 prom---->
 TAATACGACTCACTATAGGGGAATTGTGAGCGGATAACAATTCCCCTCTAGAAATAATTTTGTTTAACTTTAAGAAGGAG
 ATATACATATGGCTAGCCATCACCACCATCACCATGGCGCGAGCAAAGGAGAAGAACTCTTCACTGGAGTTGTCCCAATT
 CTTGTTGAATTAGATGGTGATGTTAACGGCCACAAGTTCTCTGTCAGTGGAGAGGGTGAAGGTGATGCAACATACGGAAA
 ACTTACCCTGAAGTTCATCTGCACTACTGGCAAACTGCCTGTTCCATGGCCAACACTAGTCACTACTCTGTGCTATGGTG
 TTCAATGCTTTTCAAGATACCCGGATCATATGAAACGGCATGACTTTTTCAAGAGTGCCATGCCCGAAGGTTATGTACAG
 GAAAGGACCATCTTCTTCAAAGATGACGGCAACTACAAGACACGTGCTGAAGTCAAGTTTGAAGGTGATACCCTTGTTAA
 TAGAATCGAGTTAAAAGGTATTGACTTCAAGGAAGATGGCAACATTCTGGGACACAAATTGGAATACAACTATAACTCAC

                                                               -----------B1MoRE-----------
 ACAACGTTCCCATCATGGCAGACAAACAAGGTGGCTCTGGCTCTGGCTCGAGTACAAGTTTGTACAAAAAAGCAGGCTCT

 --------------------------------------------->
                          <-------B2MoRE-----------------------------------
 ccgcaggacagtcgaaggtcagctgacgccctgcttaggctgcaagccatggcaggaatctcggaataataa
  P  Q  D  S  R  R  S  A  D  A  L  L  R  L  Q  A  M  A  G  I  S  E  -  -

 ------------------------
 ACCCAGCTTTCTTGTACAAAGTGGTGGATCCGGCTGCTAACAAAGCCCGAAAGGAAGCTGAGTTGGCTGCT

     <------T7 term----
 GCCACCGCTGAGCAATAACTAG
```

NGFP-100-0.6MoRE

```
--------T7 prom---->
TAATACGACTCACTATAGGGGAATTGTGAGCGGATAACAATTCCCCTCTAGAAATAATTTTGTTTAACTTTAAGAAGGAGA
TATACATATGGCTAGCCATCACCACCATCACCATGGCGCGAGCAAAGGAGAAGAACTCTTCACTGGAGTTGTCCCAATTCT
TGTTGAATTAGATGGTGATGTTAACGGCCACAAGTTCTCTGTCAGTGGAGAGGGTGAAGGTGATGCAACATACGGAAAACT
TACCCTGAAGTTCATCTGCACTACTGGCAAACTGCCTGTTCCATGGCCAACACTAGTCACTACTCTGTGCTATGGTGTTCA
ATGCTTTTCAAGATACCCGGATCATATGAAACGGCATGACTTTTTCAAGAGTGCCATGCCCGAAGGTTATGTACAGGAAAG
GACCATCTTCTTCAAAGATGACGGCAACTACAAGACACGTGCTGAAGTCAAGTTTGAAGGTGATACCCTTGTTAATAGAAT
CGAGTTAAAAGGTATTGACTTCAAGGAAGATGGCAACATTCTGGGACACAAATTGGAATACAACTATAACTCACACAACGT
TCCCATCATGGCAGACAAACAAGGTGGCTCTGGCTCTGGCTCGAGTACAAGTTTGTACAAAAAAGCAGGCTCTGATTGGGC
GAGCGGCGCGGTGAAACAGACCGCGGCGACCACCGATGATGCGACCCAGACCAGCACCACCGCGAAAGGCACCGCGACCAA
AACCGATAAAGCGACCGCGGGCGATGTGACCACCCAGAAAACCAGCCAGGCGGATGTGACCGGCGCGACCAAAACCGTGGA
TAAAGCGACCGAAAAAGTGACCAGCAAAGCGAGCACCACCACCAAACAGACCAACACCGATGGCACCACCACCGATGTGGC
GCAGACCAAACAGACCACCAGCGATACCAAAGATACCAAAGTGGATGCG

 CCGCAGGACAGTCGAAGGTCAGCTGACGCCCTGCTTAGGCTGCAAGCCATGGCAGGAATCTCGGAATAATAA
  P  Q  D  S  R  R  S  A  D  A  L  L  R  L  Q  A  M  A  G  I  S  E  -  -

 ACCCAGCTTTCTTGTACAAAGTGGTGGATCCGGCTGCTAACAAAGCCCGAAAGGAAGCTGAGTTGGCTGCT

     <------T7 term----
 GCCACCGCTGAGCAATAACTAG
```
